# Genomic and Metabolomic Analysis of the Endophytic Fungus *Alternaria alstroemeriae* S6 Isolated from *Veronica acinifolia*: Identification of Anti-Bacterial Properties and Production of Succinic Acid

**DOI:** 10.3390/antibiotics14070713

**Published:** 2025-07-16

**Authors:** Farkhod Eshboev, Alex X. Gao, Akhror Abdurashidov, Kamila Mardieva, Asadali Baymirzaev, Mirzatimur Musakhanov, Elvira Yusupova, Shengying Lin, Meixia Yang, Tina T. X. Dong, Shamansur Sagdullaev, Shakhnoz Azimova, Karl W. K. Tsim

**Affiliations:** 1 Division of Life Science and Center for Chinese Medicine R&D, The Hong Kong University of Science and Technology, Clear Water Bay, Kowloon, Hong Kong SAR, China; eshboevf@ust.hk (F.E.); gaoxiong@ust.hk (A.X.G.); lishlin@ust.hk (S.L.); mxyang@ust.hk (M.Y.); botina@ust.hk (T.T.X.D.); 2 Institute for Advanced Studies, New Uzbekistan University, Tashkent 100007, Uzbekistan; 3 S.Yu. Yunusov Institute of the Chemistry of Plant Substances, Academy of Sciences of Uzbekistan, Tashkent 100170, Uzbekistan; ahrorabdurashidov93@gmail.com (A.A.); mardievakamila@ifar.uz (K.M.); asadali.baymirzaev@gmail.com (A.B.); timur.musakhanov1202@gmail.com (M.M.); kofeechek@rambler.ru (E.Y.); icps@umail.uz (S.S.); genlab_icps@yahoo.com (S.A.)

**Keywords:** endophytic fungi, *A. alstroemeriae* S6, anti-bacterial activity, genome sequencing, AntiSMASH, succinic acid, LC-MS/MS, secondary metabolites, molecular networking

## Abstract

**Background:** Endophytic fungi are prolific sources of bioactive metabolites with potential in pharmaceutical and biotechnological applications. **Methods:** Here, the endophytic fungus, *Alternaria alstroemeriae* S6, was isolated from *Veronica acinifolia* (speedwell), and conducted its anti-microbial activities, whole-genome sequencing and metabolome analysis. **Results:** The ethyl acetate extract of this fungus exhibited strong anti-bacterial activity and the inhibition zones, induced by the fungal extract at 20 mg/mL, reached 16.25 ± 0.5 mm and 26.5 ± 0.5 mm against Gram-positive and Gram-negative bacteria. To unravel the biosynthetic potential for anti-bacterial compounds, whole-genome sequencing was conducted on *A. alstroemeriae* S6, resulting in a high-quality assembly of 42.93 Mb encoding 13,885 protein-coding genes. Comprehensive functional genome annotation analyses, including gene ontology (GO) terms, clusters of orthologous groups (COGs), Kyoto encyclopedia of genes and genomes (KEGG), carbohydrate-active enzymes (CAZymes), and antibiotics and secondary metabolites analysis shell (antiSMASH) analyses, were performed. According to the antiSMASH analysis, 58 biosynthetic gene clusters (BGCs), including 16 non-ribosomal peptide synthetases (NRPSs), 21 terpene synthases, 12 polyketide synthetases (PKSs), and 9 hybrids, were identified. In addition, succinic acid was identified as the major metabolite within the fungal extract, while 20 minor bioactive compounds were identified through LC-MS/MS-based molecular networking on a GNPS database. **Conclusions:** These findings support the biotechnological potential of *A. alstroemeriae* S6 as an alternative producer of succinic acid, as well as novel anti-bacterial agents.

## 1. Introduction

Natural compounds play a crucial role in drug discovery, particularly in fighting infectious diseases, cancers, cardiovascular diseases, and neurodegenerative diseases [1,2,3]. However, there are several challenges in growing medicinal plants, including environmental factors, pests, and diseases, as well as ethical concerns [4,5,6]. Endophytic fungi present a promising alternative in searching for active compounds; they are diverse microorganisms that reside within the internal tissues of plants, forming mutualistic relationships with the hosts [7,8,9]. These fungi can produce a wide range of structurally distinct natural compounds with significant pharmacological applications, making them essential resources in searching for new drug therapies. Fungal endophytes produce a wide range of bioactive compounds, including alkaloids, terpenoids, flavonoids, and phenolic derivatives, which exhibit anti-bacterial, anti-fungal, anti-cancer, anti-inflammatory, anti-viral, and antioxidant properties [10,11]. Indeed, a few pharmaceutical drugs derived from endophytic fungi have been developed and implemented in practice [12].

*Alternaria* is one of the most prevalent fungal genera. Being associated with plants, *Alternaria* adopts various lifestyles, including saprotrophic, endophytic, and pathogenic [13]. *Alternaria* endophytes have been shown to produce active secondary metabolites. Notable examples are camptothecin, an inhibitor of topoisomerase enzyme, produced by *A. brassicicola* isolated from *Catharanthus roseus* (periwinkle) [14]. In addition, the anti-cancer drugs, vinblastine and vincristine, derived from *Alternaria* sp., are associated with *Catharanthus roseus* [15]. Taxol, another well-known anti-cancer drug produced by *A. alternata* F3, is derived from the fruits of *Taxus cuspidate* [16]. In addition, four anti-bacterial compounds, including one novel compound, 2α-hydroxystemphytriol, and three known compounds, i.e., dihydroalterperylenol, alternariasin A, and alternariol 9-methyl ether, have been isolated from *A. alstroemeriae* derived from *C. roseus* [17]. These examples of drug discovery encourage us to reveal the genomic and metabolomic profiles of *Alternaria*.

The advancement of omics technologies provides unprecedented opportunities to unravel the genetic and biochemical basis of bioactive secondary metabolite of endophytic fungi [18]. Additionally, the antiSMASH (antibiotics and secondary metabolite shell) software has revolutionized automated genome mining for biosynthetic gene clusters (BGCs), significantly accelerating the discovery of novel natural products [19]. The genome mining of *Ascomycete* sp. F53 revealed 35 putative BGCs, leading to the identification of lijiquinone 1, a novel compound with anti-microbial and anti-cancer activities [20]. On the other hand, the *Veronica* genus, a member of the *Plantaginaceae* family, has about 500 species, and some of these species are used as medicinal plants with diverse bioactivities, including anti-bacterial, anti-tumor, antioxidant, angiogenic, and neuroprotective properties [21,22]. Here, the endophytic fungus *A. alstroemeriae* S6 was isolated from *Veronica acinifolia* (speedwell), the extract of which was probed for its anti-microbial activity. To elucidate the genetic basis of its biosynthetic pathways, we conducted whole-genome sequencing and functional annotation, revealing the key BGCs and pathways linked to the production of bioactive compounds. In addition, succinic acid was identified as the major metabolite within the *A. alstroemeriae* S6 extract, and parallelly minor bioactive compounds were revealed through LC-MS/MS-based molecular networking on the Global Natural Product Social Molecular Networking (GNPS) database. This integrative approach by combining genomics, metabolomics, and bioactivity assays provides insight into the biotechnological potential of *A. alstroemeriae* S6 as a dual-purpose platform for sustainable succinic acid production and a source of novel anti-microbial compounds.

## 2. Results

### 2.1. Anti-Bacterial Activities and Molecular Identification of A. alstromeriae S6

*A. alstromeriae* S6 was isolated from *V. acinifolia* and screened for anti-microbial activity against five pathogenic microorganisms: two Gram-positive bacteria (*B. subtilis* and *S. aureus*), two Gram-negative bacteria (*E. coli* and *P. aeruginosa*), and the yeast *C. albicans*. The ethyl acetate extracts of the fungus were evaluated using the agar disk-diffusion assay. The extract of *A. alstromeriae* S6 exhibited the strongest anti-bacterial activity, with inhibition zones of 26.5 ± 0.5 mm and 21.3 ± 0.75 mm against *B. subtilis* and *S. aureus*, respectively. For Gram-negative bacteria, the inhibition zones were measured at 16.25 ± 0.5 mm and 22.6 ± 0.5 mm for *E. coli* and *P. aeruginosa*, accordingly (Table 1). Notably, no activity was observed against *C. albicans*.

**Figure 1:**
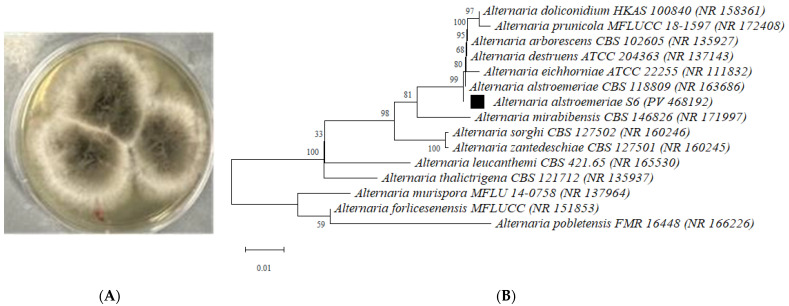
Identification of *A. alstromeriae* S6. (**A**) Colony morphology on potato dextrose agar (PDA) after 7 days of incubation at 25 °C. Petri dish diameter: 100 mm. (**B**) Neighbor-joining phylogenetic tree based on ITS region sequences, constructed using MEGA 12. Reference sequences were retrieved from GenBank (NCBI BLAST). The bootstrap consensus tree was inferred from 1000 replicates. All positions containing gaps and missing data were eliminated from the dataset (complete deletion option). Scale bar: 0.01 substitutions per nucleotide site.

The fungal isolate exhibited distinct morphological characteristics on potato dextrose agar (PDA), with initial white colonies transitioning to dark pigmentation after 7 days of incubation (Figure 1A). Molecular identification was conducted by amplifying and sequencing the ITS regions (ITS4/ITS5). The identified sequences were deposited in GenBank under accession number PV468192. BLAST analysis revealed 99.82% sequence similarity to *A. alstromeriae* strain CBS 118809 (NR163686), confirming its taxonomic classification. Phylogenetic analysis using MEGA (molecular evolutionary genetics analysis) 11 software further resolved its placement within the genius of *Alternaria*, clustering robustly with *A. alstromeriae* reference strains (Figure 1B).

### 2.2. Genome Assembly and Functional Annotation of A. alstromeriae S6

The genomic DNA of *A. alstromeriae* S6 was sequenced using a NGS (next-generation sequencing) platform (MGI). The final assembly comprised 344 contigs (N50 = 301,537 bp; largest contig = 882,804 bp) with a total length of 42.93 Mb and a GC content of 54.09%. Sequencing coverage averaged 73X across the genome, indicating a high base level of accuracy (Table 2). Genome completeness, assessed using BUSCO (benchmarking universal single-copy orthologs) v5.8.2 (fungi_odb10 dataset), revealed 99.7% completeness (99.3% single-copy, 0.4% duplicated), 0.3% missing genes, and no fragmented BUSCOs. Gene annotations using Augustus predicted 13,885 protein-coding genes, with an average protein length of 531.46 amino acids.

**Figure 2:**
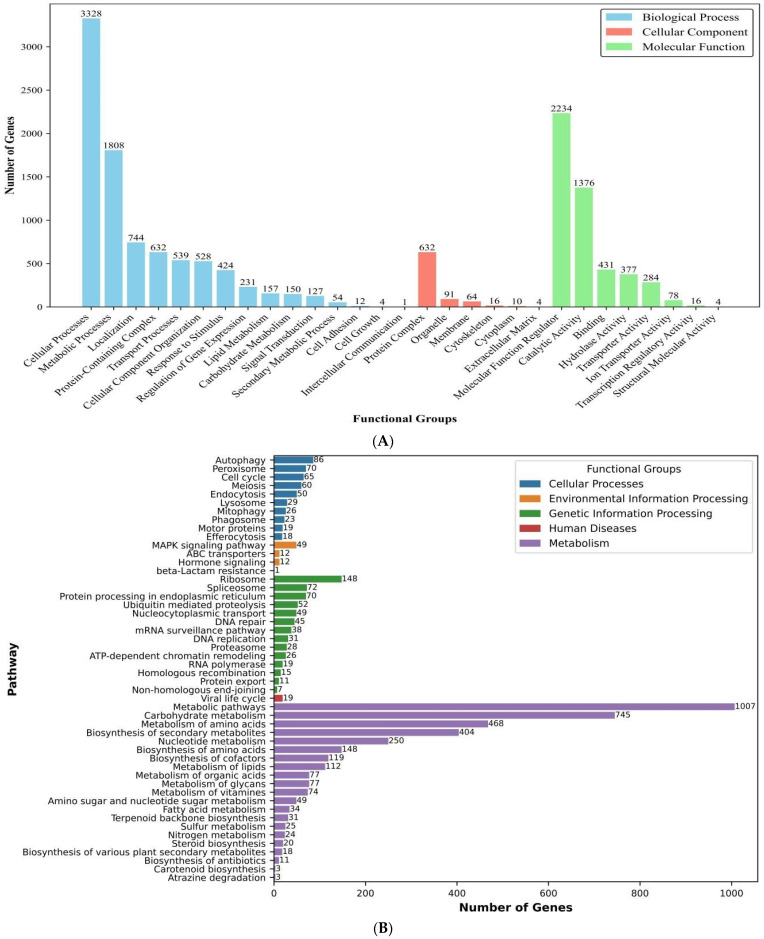
GO and COG functional annotation of *A. alstroemeriae* S6. (**A**) GO function annotation of *A. alstroemeriae* S6. GO terms were extracted from eggNOG functional annotations and InterProScan results. GO terms were mapped to hierarchical functional groups using the gene ontology database (go-basic.obo), with groups defined by user-specified parent terms. The top 15 groups were presented for each category by gene count, and terms not descending from predefined parent groups were excluded. (**B**) The COG of proteins: their function and classification. COG functional assignments were derived from eggNOG annotations using standard category descriptors, and the counts represent the total number of genes per COG category.

Functional annotation via EggNOG-mapper assigned putative function to 9303 (67%) of the predicted proteins, including GO (gene ontology) terms, and COG (collections of clusters of orthologous genes) categories. The GO analysis identified 29 functional groups categorized into three major classes: (i) biological processes, dominated by cellular processes with 3328 genes; (ii) cellular components, where protein complexes represented the largest group with 632 genes; and (iii) molecular functions, in which molecular regulators were the most abundant category, comprising 2234 genes (Figure 2A). In addition, the COG analysis classified 6549 genes (excluding 2337 function-unknown genes) into 21 functional categories, with the largest group being “general function prediction only”, which included 1239 genes, accounting for 14% of the annotated genes. Notably, 617 genes (7%) were associated with “secondary metabolite biosynthesis, transport, and catabolism” (Figure 2B).

**Figure 3:**
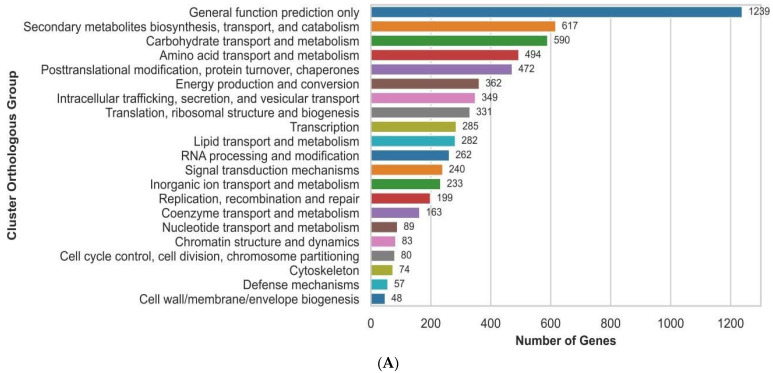

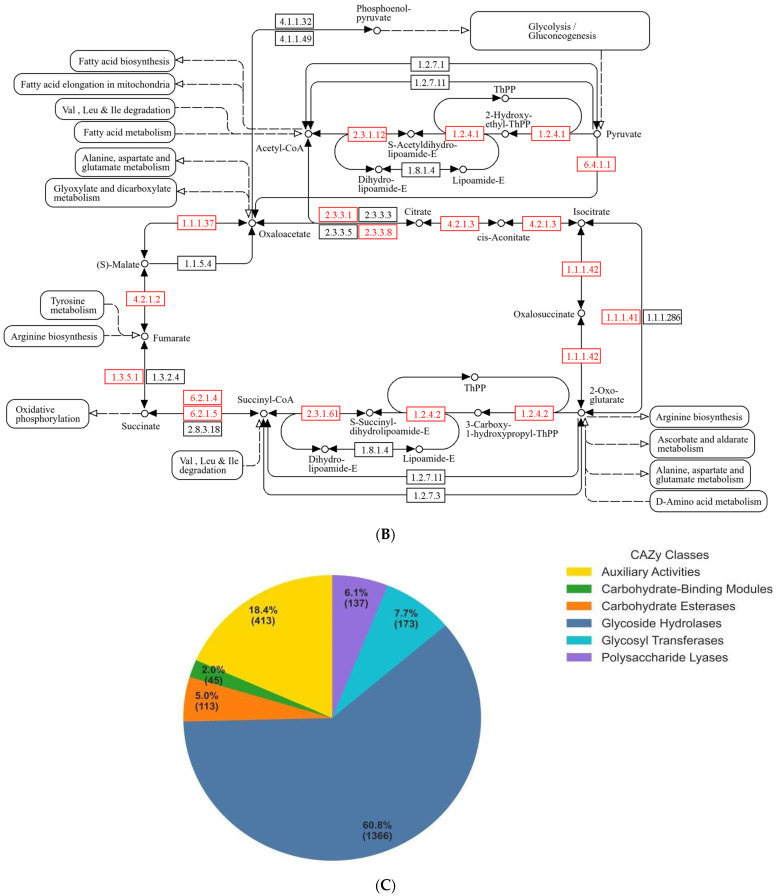
KEGG and dbCAN annotation of *A. alstroemeriae* S6. (**A**) The KEGG function annotation. KEGG pathway assignments were derived from the outputs of KEGG mapper and eggNOG. Counts represent gene assignments per pathway. (**B**) TCA cycle pathway diagram adapted from KEGG (map00020). Enzymes confirmed in *A. alstroemeriae* S6 are highlighted in red. (**C**) CAZy annotation of *A. alstroemeriae* S6. CAZy terms were extracted from dbCAN3 HMMER results and enzyme families (GH, GT, PL, CE, CBM, AA) were extracted via regex pattern matching, filtered by E-value < 1 × 10^−5^, and aggregated into functional classes with full descriptive names.

In parallel, KEGG pathway analysis mapped 4849 genes (34.9% of the proteome) to 143 metabolic pathways, distributed across 5 functional groups, i.e., cellular processes, environmental information processing, genetic information processing, human diseases, and metabolism. The most enriched pathways included carbohydrate metabolism (745 genes), amino acid metabolism (468 genes), and biosynthesis of secondary metabolites (404 genes) (Figure 3A), reflecting metabolic versatility and potential for specialized compound production of *A. alstroemeriae*. Moreover, 16 tricarboxylic acid cycle (TCA) genes (KEGG map00020) (Figure 3B) and 5 glyoxylate pathway genes (map00630) (Supplementary Figure S1) essential for succinic acid biosynthesis were identified through KEGG annotation. CAZyme (carbohydrate-active enzyme) annotation using the dbCAN database identified 3096 CAZyme domains and 455 unique genes encoding enzymes critical for carbohydrate metabolism. These were classified into six major CAZy categories: glycoside hydrolases (GHs; 1366 domains), glycosyltransferases (GTs; 173 domains), polysaccharide lyases (PLs; 137 domains), carbohydrate esterases (CEs; 113 domains), carbohydrate-binding modules (CBMs; 45 domains), and auxiliary activities (AAs; 413 domains) (Figure 3C). 

### 2.3. Secondary Metabolite Biosynthetic Potential of A. alstroemeriae S6

**Figure 4:**
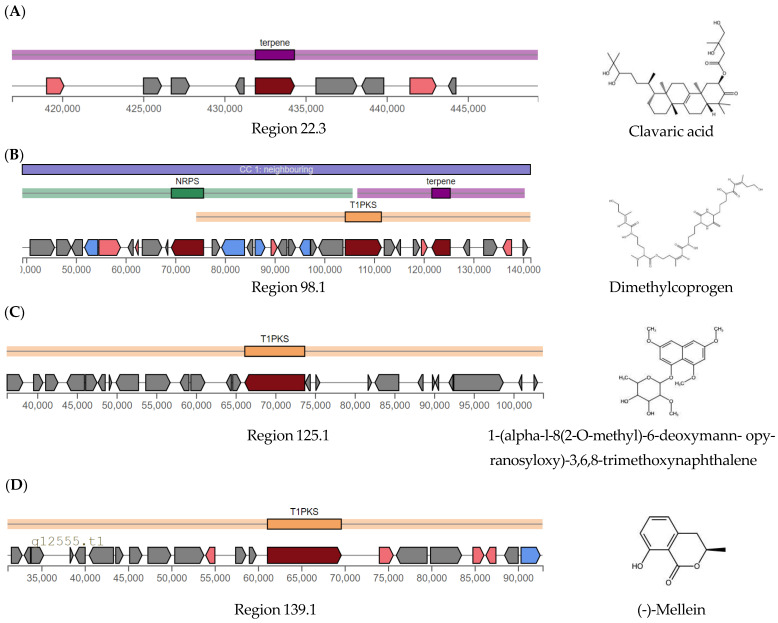

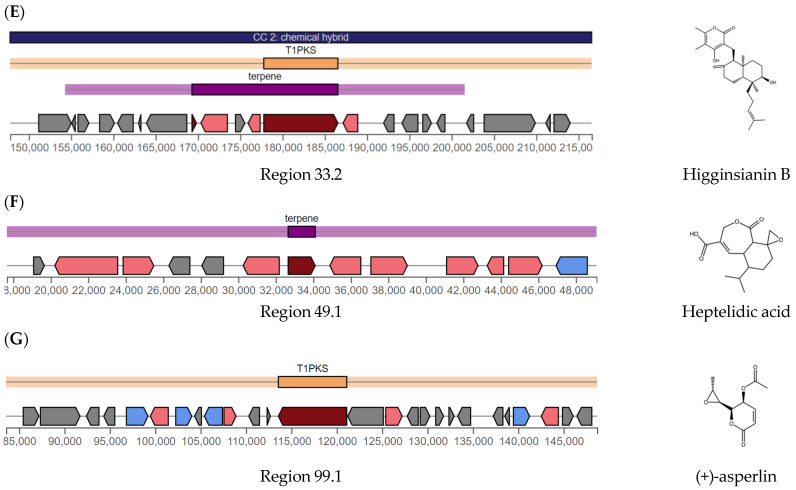
Biosynthetic gene clusters of *A. alstroemeriae* S6 identified by AntiSMASH 8.0. A–D: High-similarity clusters (green confidence level against MIBiG references). (**A**) Clavaric acid (region 22.3, node_22: 416,906–449,316 nt; 32.4 kb); (**B**) dimethylcoprogen (region 98.1, node_98: 39,203–141,477 nt; 102.3 kb); (**C**) 1-(α-l-8(2-*O*-methyl)-6-deoxymannopyranosyloxy)-3,6,8-trimethoxynaphthalene (region 125.1, node_125: 36,155–103,705 nt; 67.6 kb); (**D**) (-)-mellein (region 139.1, node_139: 31,086–92,835 nt; 61.75 kb). E–G: Medium-similarity clusters (orange confidence level against MIBiG references). (**E**) Higginsianin B (region 33.2, node_33: 147,788–216,554 nt; 68.8 kb); (**F**) heptelidic acid (region 49.1, node_49: 17,663–49,107 nt; 31.4 kb); (**G**) (+)-asperlin (region 99.1, node_99: 83,572–148,692 nt; 65.1 kb). Visualizations are generated from AntiSMASH outputs. Gene colors: core biosynthetic genes (burgundy), additional biosynthetic genes (purple), transport-related genes (blue), regulatory genes (green), and other genes (gray). Five low-similarity BGCs were excluded.

AntiSMASH analysis identified 58 biosynthetic gene clusters (BGCs) in the genome of *A. alstroemeriae* S6, categorized as follows: 16 non-ribosomal peptide synthetases (NRPSs), 21 terpene synthases, 12 polyketide synthases (PKSs), and 9 hybrid BGCs. The nine hybrid BGCs comprised four NRPS-T1PKS, three T1PKS-NRPS, one T1PKS-terpene, and one tripartite NRPS-T1PKS-terpene cluster. Comparative analysis against MIBiG (minimum information about a biosynthetic gene cluster) revealed twelve homologous BGCs in *A. alstroemeriae* S6: four high-similarity (clavaric acid, dimethylcoprogen, 1,3,5,8-tetrahydroxynapthalene, and (-)-mellein pathways) (Figure 4A–D), three medium-similarity (heptelidic acid, higginsianin B, and (+)-asperlin analogs) (Figure 4E–G), and five low-similarity (terreic acid, leucinostatin A, aspirochlorine, lucilactaene, and azanigerone A) clusters. The clavaric acid cluster, spanning 32.4 kb (region 22.3, node_22: 416,906–449,316 nt), contains nine genes, including a core triterpene synthase (g4411.t1; T2TS domain), which is flanked by oxidative tailoring enzymes (Figure 4A). A short-chain dehydrogenase/reductase (SDR) (g4407.t1) and cytochrome P450 (g4414.t1) dominate the modification machinery, exhibiting high-level homology to clavaric acid biosynthetic enzymes in *Hypholoma sublateritium* (BGC0001248.3). The P450’s conserved heme-binding motifs and the SDR’s Rossmann fold suggest their roles in C–H activation and ketoreduction, respectively, consistent with clavaric acid’s tetracyclic triterpenoid structure. Three hypothetical proteins (g4408.t1, g4409.t1, and g4413.t1) may contribute to scaffold stabilization or transport, although their exact functions remain uncharacterized.

The 102.3 kb dimethylcoprogen cluster (region 98.1, node_98: 39,203–141,477 nt) shows high homology to the characterized siderophore cluster in *Alternaria alternata* (BGC0001249.5) and represents a hybrid NRPS-T1PKS-terpene system containing 30 genes (Figure 4B). Core biosynthetic components include (1) an NRPS module (g10887.t1) featuring AMP-binding and condensation domains; (2) a type I PKS (g10898.t1) with functional KS, AT, and ketoreductase (KR) domains; and (3) a terpene synthase (g10903.t1) containing polyprenyl synthetase domains. The cluster contains five transporter genes and three cytochrome P450s, similar to the genetic structure of the siderophore pathway of *A. alternata*. Two SDRs (g10884.t1 and g10902.t1) and a siderophore biosynthesis protein (g10892.t1) further confirm its assignment as a hydroxamate-type siderophore pathway, consistent with the iron-chelating function of dimethylcoprogen.

The 67.6 kb type I PKS cluster (region 125.1, node_125: 36,155–103,705 nt) potentially governs the biosynthesis of 1-(α-l-8(2-O-methyl)-6-deoxymannopyranosyloxy)-3,6,8-trimethoxynaphthalene (Figure 4C). It features a core 6.78 kb PKS gene (g12124.t1) containing complete KS, AT, dehydratase (DH), acyl carrier protein (ACP), and enoylreductase (TE) domains, including a β-ketoacyl synthase (PF00109/PF02801) with active catalytic residues and a fungal product template domain (TIGR04532) for naphthalene formation. While the cluster shares architectural features with *Glarea lozoyensis* polyketide pathways (BGC0001258.3), it lacks identifiable glycosyltransferases for mannopyranosyl attachment, suggesting distal localization of sugar-modification genes. The compact 22-gene organization and minimal oxygenase content correspond well with the predicted trimethoxynaphthalene core structure. However, heterologous expression is required to confirm product specificity and to elucidate the missing glycosylation steps.

The (-)-mellein biosynthetic gene cluster (region 139.1, node_139: 31,086–92,835 nt) spans approximately 61.75 kb and contains 21 genes, including a core type I polyketide synthase (g12566.t1; ketosynthase (KS) and acyltransferase (AT) domains) responsible for polyketide chain assembly (Figure 4D). The key tailoring enzymes include two alcohol dehydrogenases (ADHs)—g12563.t1, zinc-dependent alcohol dehydrogenase, N-terminal domain (ADH_zinc_N), and g12570.t1, alcohol dehydrogenase, NAD(P)-binding N-terminal domain (ADH_N)—that are involved in ketoreduction. Additionally, there is a β- lactamase-like hydrolase (g12571.t1), potentially mediating lactone ring formation, and a pyridine nucleotide oxidoreductase (g12567.t1). The cluster also encodes a major facilitator superfamily transporter (g12573.t1), suggesting self-resistance mechanisms. This genetic architecture shares high synteny with the characterized (-)-mellein pathway in *Parastagonospora nodorum* (BGC0001244.3), particularly in the PKS core and reductive tailoring enzymes. The presence of dual ADHs and specialized hydrolases may reflect adaptations for (-)-mellein’s bioactive lactone structure.

Among the high-similarity clusters, (-)-mellein [23] and clavaric acid [24] are known to possess anti-bacterial (MIC 7.8–31.25 μg mL^−1^) and anti-cancer (IC50 1.3 μM) activities, respectively. The 68.8 kb higginsianin B cluster (region 2, node_33: 147,788–216,554 nt) represents a hybrid T1PKS-terpene system with medium-level similarity to *Fusarium graminearum* PH-1 (BGC0002191.2) (Figure 4E). Its core features include (i) a hybrid PKS-terpene synthase (g5909.t1) containing KS and AT domains for polyketide extension and polyprenyl synthetase motifs for isoprenoid coupling; (ii) a terpene cyclase (g5905.t1) for scaffold formation; and (iii) cytochrome P450 (g5910.t1) for oxidative tailoring. Two SDRs (g5906.t1 and g5908.t1) likely facilitate ketoreduction steps, consistent with the stereochemistry of higginsianin B.

The 31.4 kb terpenoid cluster (region 49.1, node_49: 17,663–49,107 nt), containing 13 genes, shows homology to the heptelidic acid pathway of *Aspergillus oryzae* (BGC0001995.3) (Figure 4F), which features a core terpene cyclase (g7511.t1) flanked by six cytochrome P450s (g7506.t1, g7507.t1, g7510.t1, g7512.t1, g7514.t1, g7516.t1) responsible for oxidative tailoring. The key modifications are mediated by an aldehyde dehydrogenase (g7513.t1) and short-chain dehydrogenases/reductases (SDRs; g7515.t1), while a sugar transporter (g7517.t1) suggests the export of products.

The (+)-asperlin biosynthetic gene cluster at 65.1 kb (region 99.1, node_99: 83,572–148,692 nt) contains 26 genes, including a core type I polyketide synthase (g10950.t1) with KS, AT, KR, and ER domains required for polyketide assembly (Figure 4G). Two cytochrome P450 genes (g10952.t1 and g10961.t1) likely mediate oxidative modifications, while four transporter genes (g10942.t1, g10944.t1, g10946.t1, and g10960.t1) suggest the self-resistance mechanisms. Additional biosynthetic genes include an amidase (g10943.t1) and an iron-dependent oxygenase (g10947.t1), which may contribute to tailoring reactions. The cluster shares functional similarities with prolipyrone B- and gibepyrone D-producing systems of *Fusarium graminearum* (BGC0002191.2), although direct homologs were not detected in MIBiG.

Amid medium-similarity compounds, heptelidic acid [25] and higginsianin B [26] are well known to have anti-bacterial (inhibition zone 15 mm) and anti-cancer (IC50 1.0 μM) activities, accordingly. The 46 unidentified BGCs (79.3%) position *A. alstroemeriae* S6 as a candidate for biosynthesis of new natural products against drug-resistant pathogens through heterologous expression and metabolomic mining.

### 2.4. Natural Product Isolation and Metabolomic Profiling

**Figure 5:**
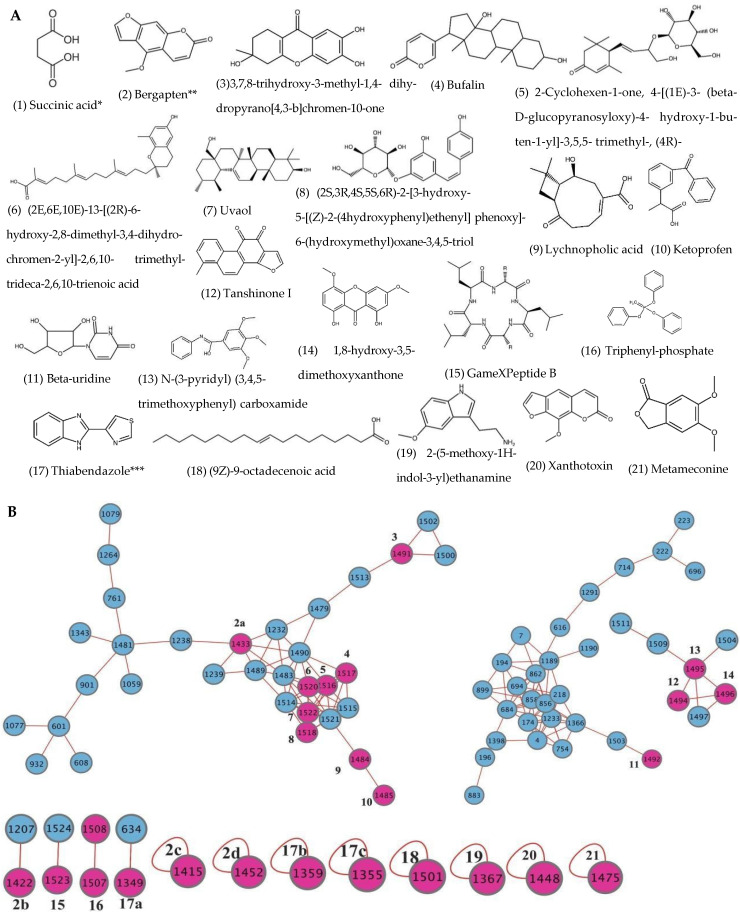
Molecular network of *A. alstroemeriae* S6 secondary metabolites. (**A**) Structures of 21 identified compounds. (*) Succinic acid (1) was isolated and structurally confirmed by X-ray crystallography; (**) bergapten (2) appeared in 4 nodes (2a–2d); (***) thiabendazole (17) occurred in 3 nodes (17a–17c). (**B**) Network displays 15 compounds (highlighted in red) containing nodes out of 77 total nodes. Numbers within nodes indicate cluster assignments and edges represent spectral similarity (cosine score ≥ 0.6). Molecular network analysis was performed using GNPS with MS/MS spectral data, and the network was visualized using Cytoscape 3.10.3.

Following ethyl acetate extraction of the fungal culture, a substantial quantity of crystalline material was observed adhering to the inner surfaces of the glass dish. To identify the major crystalline compound, the crystals were purified through recrystallization and sequential washes with organic solvents. Single-crystal X-ray diffraction identified the compound as succinic acid (Figure 5A) [27]. The isolated crystals accounted for 55% (4 g/L fungal culture) of the total dry extract weight, highlighting *A. alstroemeriae* S6 as a promising candidate for biotechnological production of succinic acid due to its high yield and easy purification. To characterize the remaining part of the extract, LC-MS/MS analysis coupled with GNPS tentatively identified another 20 secondary metabolites (Figure 5A). The identified metabolites comprised five terpenoids (bufalin, lychnopholic acid, tanshinone I, uvaol, and (2E,6E,10E)-13-[(2R)-6-hydroxy-2,8-dimethyl-3,4-dihydrochromen-2-yl]-2,6,10-trimethyl-trideca-2,6,10-trienoic acid); four alkaloids (N-(3-pyridyl)(3,4,5-trimethoxyphenyl) carboxamide, β-uridine, thiabendazole, and 2-(5-methoxy-1H-indol-3-yl)ethanamine); two furocoumarins (bergapten and xanthotoxin); two polyketides (metameconine and 1,8-dihydroxy-3,5-dimethoxyxanthone); one phenolic glycoside ((2S,3R,4S,5S,6R)-2-[3-hydroxy-5-[(Z)-2-(4-hydroxyphenyl)ethenyl]phenoxy]-6-(hydroxymethyl)oxane-3,4,5-triol); one fatty acid ((9Z)-9-octadecenoic acid); one cyclic peptide (GameXPeptide B); and two pharmaceutical residuals (ketoprofen and triphenyl-phosphate). Mass accuracy assessment classified 14 compounds as high-confidence identifications (≤5 ppm mass error) and 6 as medium-confidence (5–10 ppm mass error) based on precursor ion alignment.

The molecular network was visualized based on GNPS output data using Cytoscape 3.10.3. It revealed 77 nodes organized into 1525 spectral clusters. The network topology showed three distinct tiers of metabolite distribution (Figure 5B): (i) a dominant cluster of 33 interconnected nodes containing 9 identified compounds (Figure 5A: compounds **2**–**10**); (ii) intermediate nodes comprising 27 nodes (β-uridine) and 7 compounds (compounds **12**–**14**), either extensive structural variants or limited dereplication; and (iii) peripheral features including 4 dimeric nodes (2 nodes each) containing compounds **15**–**17**, along with 8 singleton nodes. Notably, bergapten and thiabendazole appeared in four and three nodes, respectively, suggesting either multiple derivative forms or analytical artifacts. The network highlights both the chemical diversity of the fungus (spanning terpenoids, furanocoumarins, and unusual metabolites like the cyclohexanone derivative) and the challenges of dereplication, where the dominant clusters may represent core metabolic pathways while singletons could indicate rare or novel chemistry.

Among the determined compounds, four compounds have potential anti-bacterial activities, according to the literature. Bergapten (C_12_H_8_O_4_) is a natural furanocoumarin widely found in medicinal plants, and which has various biological properties, including anti-microbial, anti-cancer, neuroprotection, anti-inflammatory, and anti-diabetes effects [28]. Zaher et al. [29] determined that the production of bergapten could be generated from endophytic fungus *Botryodiplodia theobromae* isolated from *Dracaena draco*, and this compound showed strong anti-bacterial activities. Lychnopholic acid (C_15_H_22_O_3_) was isolated from *Lychnophora affinis* and *Lychnophora salicifolia,* showing strong anti-microbial activities against *C. albicans*, *E. coli*, and *S. aureus*, as well as anti-tumor properties [30,31].

GemeXpeptideB (C_32_H_51_N_5_O_5_) is a cyclic non-ribosomal homologous peptide, known as an anti-bacterial peptide, synthesized by entomopathogenic bacterium *Xenorhabdus nematophilus* [32]. Xantotoxin (C_12_H_8_O_4_) is a natural linear furanocoumarin widely distributed in plants and found in small amounts at microbial sources. Xantotoxin exhibits a broad range of pharmacological activities, including anti-bacterial, neuroprotective, skin-repairing, anti-inflammatory, antioxidant, and insecticidal effects [33]. In addition to anti-bacterial compounds, bufalin (C_24_H_34_O_4_) [34] and tanshinone I (C_18_H_12_O_3_) [35], considered as anti-cancer drugs, were also identified in the extract. These results demonstrate that *A. alstroemeriae* S6 possesses significant potential in the biosynthesis of diverse bioactive secondary metabolites. However, the compounds predicted by GNPS (Global Natural Products Social Molecular Networking) showed limited overlap with those associated with antiSMASH-identified BGCs. Only bergapten and xanthotoxin were aligned genomically, originating from the T4HN-type PKS cluster (BGC0001548) via conserved fungal furanocoumarin pathways. The remaining 17 metabolites, including all 5 terpenoids, showed no genomic correlation despite the presence of 21 terpene synthases, implying there are silent biosynthetic clusters or non-canonical pathways that require further activation studies. These findings support the capacities of *A. alstroemeriae* to synthesize diverse bioactive metabolites with anti-bacterial and/or anti-cancer properties. 

## 3. Discussion

This work demonstrates that the endophytes of medicinal plants are valuable targets in discovering novel natural products, and which can serve as alternative producers of biotechnologically important compounds, such as succinic acid and anti-bacterial agents. Here, we isolated endophytic fungi from *V. acinifolia,* a previously unexplored host, and evaluated their anti-microbial activities. Indeed, *Veronica* species are commonly recognized as suitable hosts for endophytic fungi [36], and this is the first report of fungal isolation from *V. acinifolia*. Among the isolated fungi, the extract of *A. alstroemeriae* S6 exhibited the highest anti-bacterial activity. Consequently, the subsequent investigations, including taxonomic identification, whole-genome sequencing, and secondary metabolite profiling of the fungus, aimed to elucidate its biosynthetic potential. The fungal isolate was identified as *A. alstroemeriae* through ITS region sequencing and BLAST analysis against the NCBI database. *A. alstroemeriae* is primarily recognized as a fungal pathogen. These endophytic strains have been isolated and have been shown to produce bioactive compounds [37]. For example, the endophytic fungus *A. alstroemeriae* was isolated from the medicinal plant *Artemisia artemisia*, and the crude extracts of this fungus demonstrated strong inhibition on cultured A549 tumor cells [38]. However, the anti-cancer properties of the extracts of *A. alstroemeriae* S6 have not been evaluated here. In parallel, the ethyl acetate extract of the endophytic fungus *A. alstroemeriae,* isolated from *Fagopyrum dibotrys,* showed strong anti-bacterial activities against *E. coli* and *S. aureus* [39].

Genome-mining-based strategies offer new insights to discover novel natural compounds, as compared to the conventional bioactivity-guided screening approaches [40]. To elucidate the genomic determinants of anti-bacterial activity in *A. alstroemeriae* S6, we performed whole-genome sequencing, revealing a 42.93 Mb genome encoding 13,885 proteins. Notably, only one other *A. alstroemeriae* genome has been isolated from *Gallus gallus* (GenBank: GCA_037044435.1) with a genome size of 35.5 Mb (https://www.ncbi.nlm.nih.gov/datasets/genome/GCA_037044435.1/ accessed on 25 March 2025). The observed differences in genome size (42.93 Mb vs. 35.5 Mb) could be due to the specialization in ecological niche, as endophytic fungi often harbor expanded BGCs for host interactions and environmental competition [41,42]. Moreover, the number of protein-encoding genes can be different among various *Alternaria* species. For example, Tao et al. [43] have described the number of protein-encoding genes in different *Alternaria* species that could range between 9789 and 24,347. While the absence of a high-quality reference genome for *A. alstroemeriae* limits strain-specific comparisons, our assembly provides a foundation for future studies of its genomic architecture.

After genome assembly, the functional annotation using eggNOG mapper was performed by using the results of Augustus. Functional annotation of the *A. alstroemeriae* S6 genome highlighted its exceptional capacity for biosynthesis of secondary metabolites. GO analysis has linked 54 functional groups to secondary metabolic processes, including specialized pathways for the synthesis of anti-microbial compounds. COG annotation identified 617 genes directly associated with secondary metabolite biosynthesis, transport, and catabolism, emphasizing the genetic basis of the strain to produce chemical compounds. KEGG pathway mapping resolved 404 and 11 genetic pathways responsible for the biosynthesis of secondary metabolites and antibiotics, respectively. These findings support *A. alstroemeriae* S6’s versatility in producing diverse secondary metabolites, enabling ecological competition in its host environment and offering biotechnological potential for producing novel bioactive compounds. In addition, the identification of all essential genes for the biosynthesis of succinic acid in both TCA cycle and glyoxylate pathways provides genomic evidence to support the production capability of *A. alstroemeriae* S6. Furthermore, genome editing, e.g., CRISPR-Cas9 targeting succinate dehydrogenase, could further enhance the yield of succinic acid by optimizing metabolic flux through these pathways.

CAZymes are important enzymes for carbohydrates metabolism of fungi [44]. Therefore, we performed CAZyme annotation of *A. alstroemeriae* S6 on the dbCAN3 web server, and 455 unique genes responsible for carbohydrate-active enzymes were detected. Comparative analysis revealed that *A. alstroemeriae* S6 encodes fewer CAZymes than other *Alternaria* species, such as *Alternaria* sp. SPS-2, isolated from *Echrysantha chrysantha* Lindl., which harbored 644 CAZyme-encoding genes, suggesting the niche-specific adaptations in carbohydrate metabolism [43]. The abundance of glycoside hydrolases (1366 domains) reflects adaptations to plant biomass utilization, while auxiliary activities (413 domains), such as AA9 family lytic polysaccharide monooxygenase, suggest oxidative lignin modification capabilities, underscoring a dual strategy for carbohydrate metabolism and ecological niche specialization of *A. alstroemeriae* S6.

The biosynthesis of bioactive secondary metabolites in endophytic microorganisms is regulated by specialized gene clusters that encode enzymes for their synthesis, modification, and transport. Coordinated expression of these genes produces structurally complex compounds with diverse bioactivities [45]. Tools like antiSMASH leverage computational algorithms and databases to predict these clusters, enabling the identification and optimization of metabolite production [46,47]. Here, antiSMASH analysis of *A. alstroemeriae* S6 identified 58 BGCs. Comparative analysis against the MIBiG repository revealed 12 BGCs with varying levels of similarity and 46 BGCs (79%) showing no homology to known pathways, suggesting a substantial novelty. The dominance of terpene (21 BGCs) and NRPS (16 BGCs) clusters aligns with anti-microbial activities of the strain. However, the disparity between antiSMASH predictions and metabolomic profiles highlights the need for activation strategies, such as epigenetic induction, to unlock its full biosynthetic capabilities. These findings position *A. alstroemeriae* S6 as a promising candidate for anti-microbial drug discovery and evolutionary studies of fungal secondary metabolism.

Succinic acid was determined to be the dominant metabolite produced by *A. alstroemeriae* S6, comprising 55% of the dried weight of ethyl acetate extract. Succinic acid is extensively utilized in pharmaceuticals, functional foods as flavor enhancers, and biodegradable plastics [48]. Current production methods primarily depend on petrochemical-based synthesis, which is environmentally unfriendly, or on bacterial and yeast fermentation [49,50]. In this context, *A. alstroemeriae* S6 presents a sustainable alternative for the production of succinic acid. The growth of *A. alstroemeriae* S6 offers distinct advantages, including utilization of low-cost substrates, natural tolerance to fermentation inhibitors, and reduced regulatory hurdles as a non-engineered organism. Future studies focusing on the process of optimization and strain adaptation could increase the yield of the acid. The ecological role of succinic acid in *A. alstroemeriae* S6 remains speculative, but it could be responsible for pH regulation or stress response in its host, i.e., *V*. *acinifolia*. Succinic acid is a tricarboxylic acid (TCA) cycle intermediate in cells; its extracellular accumulation in such quantities suggests an overflow metabolism under nutrient-limited conditions, a phenomenon observed commonly in other fungi [51]. Focusing on succinic acid as a primary metabolite departs from the typical emphasis on fungal secondary metabolites, e.g., polyketides and terpenes. This highlights the dual biotechnological value of *A. alstroemeriae* S6 as a high-value platform to generate chemicals and bioactive secondary metabolites. The fermentation optimization, including carbon source modulation and the activation of silent clusters, can improve the yield of succinic acid while uncovering cryptic secondary metabolites. Such efforts could bridge the gap between microbial ecology and industrial biotechnology, leveraging endophytic fungi for sustainable chemical production. Moreover, the strong anti-bacterial activity of the extract of *A. alstroemeriae* S6 could be linked to its high content of succinic acid. Indeed, succinic acid exhibited significant anti-bacterial effects, with inhibition zones of 27.18 ± 0.16 mm against *S. aureus* and *P. fluorescens* with inhibition zones of 27.18 ± 0.16 mm and 18.98 ± 0.62 mm, respectively [52].

The LC-MS/MS and GNPS analyses of the extract of *A. alstroemeriae* S6 tentatively identified twenty secondary metabolites, including four anti-bacterial compounds, bergapten, lychnopholic acid, GemeXpeptideB, and xantotoxin, as well as anti-cancer agents bufalin and tanshinone I. Additionally, the anti-inflammatory compounds, such as uvaol [53], piceid [54], and ketoprofen [55], were detected in the extract, broadening the pharmacological potential of *A. alstroemeriae* S6. These findings support the biosynthetic richness of *A. alstroemeriae* S6 and highlight the need for additional studies to review the chemical nature of anti-cancer and anti-inflammatory activities. However, the limited overlap between GNPS metabolites and antiSMASH-predicted BGCs suggests that most clusters remain silent under standard conditions or are not able to be detected due to insufficient database coverage. Thus, further research is required for individual isolation, structure elucidation, and quantification of these natural compounds.

## 4. Materials and Methods

### 4.1. Bacterial Strains

The microorganisms, *Bacillus subtilis* RKMUz-5, *Escherichia coli* RKMUz-221, and *Candida albicans* RKMUz-247, were obtained from the collection of microorganisms of the Institute of Microbiology, Academy of Sciences of Uzbekistan, and the pathogenic bacteria, *Staphylococcus aureus* ATCC 25923 and *Pseudomonas aeruginosa* ATCC 27879, were from the American Type Culture Collection (ATCC).

### 4.2. High-Performance Computing Setup

All bioinformatics analyses associated with genome assembly and annotation were conducted on the HPE ProLiant XL170r Gen10 high-performance computing (HPC) system with Python version 3.8.20. and Linux-3.10.0–1062.el7.x86_64-x86_64-with-glibc2.17.

### 4.3. Isolation of Endophytic Fungi

The plants, V. acerifolia, were collected from the Botanical Garden of the Academy of Sciences of Uzbekistan (41.3448° N, 69.3107° E), transported to the laboratory, and immediately processed for endophytic fungal isolation, as described previously [8]. Briefly, the stems and leaves of the plants were washed in sterile water and surface-sterilized in 4% sodium hypochlorite for 1 min, followed by 70% ethanol for 1 min and 1% sodium hypochlorite for 2 min, and were then washed in sterile distilled water. The surface-sterilized plant materials were aseptically cut into small fragments and directly placed on potato dextrose agar (PDA) medium, supplemented with 100 mg/L ampicillin, and incubated at 25 °C for 2 weeks. To test the efficacy of surface sterilization, 200 µL of water from the final washing stage was additionally inoculated onto PDA and incubated at 25 °C. After incubation, the fungal colonies with different morphologies were cultivated on PDA media and incubated for a week. To obtain the final pure cultures, the isolates were cultured several times on PDA.

### 4.4. Extraction of Fungal Secondary Metabolites

The fungal strain was cultivated in 500 mL Erlenmeyer flasks, each containing 200 mL of potato dextrose broth (PDB). The flasks were incubated on a rotary shaker at 28 °C and 150 rpm for 14 days. After the incubation period, the fungal cultures were subjected to liquid–liquid extraction five times with an equal volume of ethyl acetate. The combined extracts were concentrated under reduced pressure at 40 °C using a rotary evaporator (Heidolph Hei-VAP) to yield the dried crude extract [8].

### 4.5. Anti-Microbial Activity of the Extract of Fungus

The anti-bacterial activity of ethyl acetate extracts, derived from *V. acerifolia*-associated endophytic fungi, were evaluated using the agar disk-diffusion method [56,57]. Anti-microbial efficacy was assessed against five pathogenic strains: *B. subtilis* RKMUz-5, *S. aureus* ATCC 25923, *E. coli* RKMUz-221, *P. aeruginosa* ATCC 27879, and *C. albicans* RKMUz-247. The microorganism cells (200 µL) were added to 25 mL nutrient agar and Mueller–Hinton agar for bacterial and *C. albicans*, respectively. The dried fungal extracts were dissolved with methanol (at 20 mg/mL) and applied onto the agar plate using sterile filter paper disks. The disks were completely air-dried in a laminar flow hood until all solvents evaporated. Ampicillin/sulbactam (10 µg/disk each), gentamicin (10 µg/disk), and fluconazole (25 µg/disk) were used as positive controls. Solvent-treated disks served as negative controls. The plates were incubated at 37 °C for 24 h (bacterial strains) or 28 °C for 48 h (*C. albicans*). After incubation, the diameter of inhibition zone was measured.

### 4.6. Identification of Endophytic Fungi

The identification of the fungus was conducted based on sequencing of the internal transcribed spacer (ITS) region of the ribosomal gene. Genomic DNA was extracted from fresh fungal culture using the E.Z.N.A. Tissue DNA kit (Omega Bio-tek, Norcross, GA, USA) with a minor modification of the lysis step involving 60 min of incubation at 95 °C. PCR amplification was performed in 25 µL reactions containing 2× EmeraldAmp^®^ GT PCR Master Mix (Takara Biomedical Technology, Beijing, China), 10 µL of gDNA, and primers ITS5 (forward: 5′ GGA AGT AAA AGT CGT AAC AAG G 3′) and ITS4 (reverse: 5′ TCC TCC GCT TAT TGA TAT GC 3′) [58]. The PCR conditions were 5 min at 95 °C; 35 cycles of 15 s at 95 °C, 10 s at 58 °C, and 30 s at 72 °C; and final extension at 72 °C for 5 min. The PCR products were visualized on 1% agarose gel and purified by ethanol precipitation. Sanger sequencing was performed by Tech Dragon Ltd. (Hong Kong, China) using the ITS5/ITS4 primers. Sequences were deposited in the NCBI GenBank database. Phylogenetic analysis was conducted in MEGA 11 using the neighbor-joining method with 1000 bootstrap replicates after alignment via MUSCLE [59].

### 4.7. Genome Sequencing and Assembly

The isolate S6 was cultured in potato dextrose broth (PDB) medium at 28 °C for 48 h. Genomic DNA was extracted as described above, and its concentration and purity were assessed using a NanoDrop 2000 spectrophotometer (Thermo Fisher Scientific, Waltham, MA, USA) and Qubit^®^ 4.0 Fluorometer with the dsDNA HS Assay Kit (Thermo Fisher Scientific). Sequencing libraries were prepared using the MGI Easy FS DNA library preparation kit (V2.1) and subjected to whole-genome sequencing on an MGI DNBSEQ-T7 platform (MGI Tech, Hong Kong, SAR, China) at the Bio-CRF facility, Hong Kong University of Science and Technology (HKUST). Raw paired-end reads were quality-checked using FastQC (v0.11.9) (https://www.bioinformatics.babraham.ac.uk/projects/fastqc/ (accessed on 25 March 2025)), and adapter sequences were trimmed with Trimmomatic (v0.39) [60]. In the absence of a closely related reference genome, we employed *de novo* assembly to avoid reference bias. Processed reads were converted to Illumina-compatible FASTQ format and assembled *de novo* with SPAdes (v3.15.5) under the “isolate” mode [61]. K-mer sizes were iteratively optimized as 21, 33, 55, 77, 99, and 127. Assembly quality was evaluated using QUAST (v5.3.0) [62], and genome completeness was assessed with BUSCO (v5.8.1) against the fungi_odb10 database (January 2024 release; 758 BUSCOs from 549 fungal genomes) [63]. The whole-genome data have been deposited in the National Center for Biotechnology Information database (www.ncbi.nlm.nih.gov) under the BioProject number PRJNA1248411.

### 4.8. Gene Prediction and Annotation

Protein-coding genes were predicted from the assembled genome using Augustus (v3.5.0) [64] by using the known sequences from *Botrytis cinerea*. InterProScan (v5.59–91.0) [65] was used to identify protein domains, motifs, and GO terms by scanning against integrated databases (Pfam, PROSITE, PRINTS, SMART, PANTHER, and SUPERFAMILY). Functional annotation of the predicted genes was performed using eggNOG-mapper (v2.1.12) [66] with default parameters. The eggNOG database (v5.0.0) [67] was employed, which includes 5090 organisms, 4.4 million orthologous groups, and associated phylogenetic trees and multiple sequence alignments. Annotations included GO terms, COG categories, and KEGG pathways. KEGG Automatic Annotation Server (KAAS) [68] provided pathway annotations using the bi-directional best-hit method against the KEGG GENES database (release 1 July 2023). CAZymes were classified using the dbCAN3 meta server with HMMER (v3.4.0) against the CAZy database (v13.0, released 9 July 2024) [69]. The analysis utilized dbCAN3 HMMdb v13 (released 14 August 2024), which included 826 CAZyme hidden Markov models (HMMs) and aligned with the updated CAZy database.

### 4.9. Identification of Secondary Metabolite Biosynthetic Clusters

Secondary metabolite BGCs were identified using the antiSMASH fungal web server (version 8.0 beta) with default parameters [70]. The analysis included detection of core biosynthetic genes, tailoring enzymes, and cluster boundary prediction. Identified BGCs were compared with the minimum information about a biosynthetic gene cluster (MIBiG) database (V4.0) using BLASTP to annotate the known metabolite classes [71].

### 4.10. Purification and Structure Elucidation of Succinic Acid

The ethyl acetate-soluble fraction of the fungal crude extract was subjected to solvent-assisted crystallization for purification of succinic acid. Briefly, the extract was dissolved in a minimal volume of warm methanol, and n-hexane was gradually added until cloudiness persisted. The mixture was allowed to stand at 4 °C for 24 h, yielding colorless crystalline precipitates. The crystals were collected via vacuum filtration, washed sequentially with cold n-hexane and diethyl ether to remove impurities, and recrystallized twice in methanol to improve the purity level. The structure of the purified succinic acid was determined by single-crystal X-ray diffraction analysis using a Bruker D8 VENTURE diffractometer (Bruker, Germany).

### 4.11. LC-MS/MS Analysis

Liquid chromatography–tandem mass spectrometry (LC-MS/MS) analysis of the crude extract of isolate S6 was performed using an Orbitrap Exploris 120 mass spectrometer (Thermo Scientific). Chromatographic separation was carried out through an ACE Excel Super C18 column (250 × 4.6 mm; 5 µm) with mobile phases: A (0.1% formic acid in water) and B (0.1% formic acid in methanol). The gradient elution program was as follows: 0–1 min: 5% B (isocratic); 1–11 min: 5–100% B (linear gradient); 11–13 min: 95% B (isocratic); 13–15 min: 5% B (linear gradient); and 15–16 min: 5% B (column equilibration). The analysis was conducted in positive ionization mode (H-ESI) with the following parameters: static spray voltage; sheath gas: 35 arbitrary units (Arb); auxiliary gas: 10 Arb; ion transfer tube temperature: 270 °C; and vaporizer temperature: 375 °C. Full-scan MS spectra (resolution: 60,000) were acquired over *m*/*z* 100–1600, followed by data-dependent MS/MS scans (resolution: 15,000) of the top 4 precursor ions. Raw data files were converted to mzML format using MSConvert (ProteoWizard) and analyzed with MZmine3 (v4.4.3) [72]. The mass spectrometry data have been deposited on the Global Natural Products Social Molecular Networking (GNPS) web platform under the accession number MassIVE ID: MSV000097857 for molecular networking [73]. The network was visualized using Cytoscape (v3.10.3) [74], and metabolite annotations were assigned based on spectral library comparisons.

## 5. Conclusions

Endophytic fungi represent a vital source of industrially valuable natural compounds. In this study, *A. alstroemeriae* S6 was isolated from *V. acinifolia*, growing in Uzbekistan, and the extract of the isolate exhibited strong anti-bacterial activity. Our whole-genome sequencing of *A. alstroemeriae* S6, the second report for this species in the NCBI database, yielded a high-quality assembly revealing 13,885 protein-coding genes. Comprehensive functional annotations, including GO, COG, KEGG, CAZymes, and antiSMASH analysis, were performed to elucidate the genetic basis of the morphological, physiological, and biosynthetic traits of *A. alstroemeriae* S6, particularly its production of secondary metabolites. The current result has proposed a new producer of succinic acid, yielding 4 g/L, from the fungus. Additionally, AntiSMASH identified 58 BGCs, predominantly terpene and NRPS clusters, while LC-MS/MS raw-data-based molecular networking on the GNPS database identified 20 bioactive metabolites, including anti-bacterial compounds (bergapten, lychnopholic acid, GemeXpeptideB, and xantotoxin), anti-cancer agents (bufalin and tanshinone I), and anti-inflammatory compounds (uvaol, piceid, and ketoprofen). The isolated fungus *A. alstroemeriae* S6 can serve as a dual-purpose platform for chemical production: a sustainable producer of succinic acid and a reservoir of bioactive natural compounds. Future work should prioritize mechanistic studies of the secondary metabolites of *A. alstroemeriae* S6 against antibiotic-resistant pathogens, as well as its anti-cancer and anti-inflammatory effects. Additionally, the fermentation optimization for metabolite scale-up and genome-editing approaches could be able to unlock the silent BGCs.

## Figures and Tables

**Table 1:** Anti-microbial activity of ethyl acetate extract from *A. alstroemeriae* S6.

Samples	Inhibition Zone (Mean ± SE, *n* = 3) ^a^				
	Gram-Positive Bacteria		Gram-Negative Bacteria		Fungus
	*B. subtilis*	*S. aureus*	*E. coli*	*P. aeruginosa*	*C. albicans*
EtOAc extract ^b^	26.5 ± 0.5	21.3 ± 0.75	16.25 ± 0.5	22.6 ± 0.5	Not active ^c^
Ampicillin ^d^	30.4 ± 0.15	26.45 ± 0.2	Not tested	Not tested	Not tested
Gentamicin	Not tested	Not tested	25.15 ± 0.25	26.5 ± 0.3	Not tested
Fluconazole	Not tested	Not tested	Not tested	Not tested	33.0 ± 0.2

^a^ Inhibition zones measured by agar diffusion assay in mm. ^b^ Ethyl acetate (EtOAc) extract of *A. alstroemeriae* S6 at 20 mg/mL. ^c^ “Not active” indicates no detectable inhibition zone. ^d^ Positive controls: ampicillin (10 μg/disk); gentamicin (10 μg/disk); fluconazole (25 μg/disk).

**Table 2:** Genome assembly statistics for *A. alstroemeriae* S6 based on whole-genome sequencing.

Genome Features	Value
Sequencing coverage ^a^	73×
Total assembly size (Mb) ^b^	42.93
Number of contigs ^c^	344
Largest contig ^d^	882,804
N50 ^e^	301,538
N90 ^f^	78,886
L50 ^g^	40
L90 ^h^	145
GC content ^i^ (%)	54.09
BUSCO ^j^ (%)	99.7
Protein-coding genes ^k^	13,885

^a^ Average depth of sequencing across the genome; ^b^ complete size of the assembled genome; ^c^ total contiguous sequences in the assembly; ^d^ size of the longest continuous DNA segment; ^e^ contig length, where 50% of the total assembly is contained in contigs of this size or larger; ^f^ contig length covering 90% of the assembly; ^g^ number of contigs that collectively cover half the assembly; ^h^ number of contigs covering 90% of the assembly; ^i^ percentage of guanine–cytosine base pairs in the genome; ^j^ completeness assessment against conserved orthologs; ^k^ predicted functional genes in the assembly.

## Data Availability

The complete genome sequence data have been deposited at GenBank (BioProject: PRJNA1248411). The mass spectrometry data have been deposited on GNPS under the accession number MassIVE ID: MSV000097857.

## Supplementary Materials

The following supporting information can be downloaded at https://www.mdpi.com/article/10.3390/antibiotics14070713/s1, Figure S1: Glyoxylate pathways diagram adapted from KEGG (map00630); Table S1: eggNOG-mapper annotations;.

## Abbreviations

The following abbreviations are used in this manuscript:

BLAST	Basic Local Alignment Search Tool
GO	Gene Ontology
KEGG	Kyoto Encyclopedia of Genes and Genomes
COG	Cluster of Orthologous Groups
CAZyme	Carbohydrate Activity Enzyme
antiSMASH	Antibiotics and Secondary Metabolite Analysis Shell
BGCs	Biosynthetic Gene Clusters
GNPS	Global Natural Products Social Molecular Networking
