# Supplementary material for: Genomic and Metabolomic Analysis of the Endophytic Fungus Alternaria alstroemeriae S6 Isolated from Veronica acinifolia: Identification of Anti-Bacterial Properties and Production of Succinic Acid
Source: Antibiotics (Basel). 2025 Jul 16;14(7):713. doi: 10.3390/antibiotics14070713 (PMC12291967; doi:10.3390/antibiotics14070713)
Supplement: Supplementary file 1 [file antibiotics-14-00713-s001.zip › Supplementary figure S1.pdf]

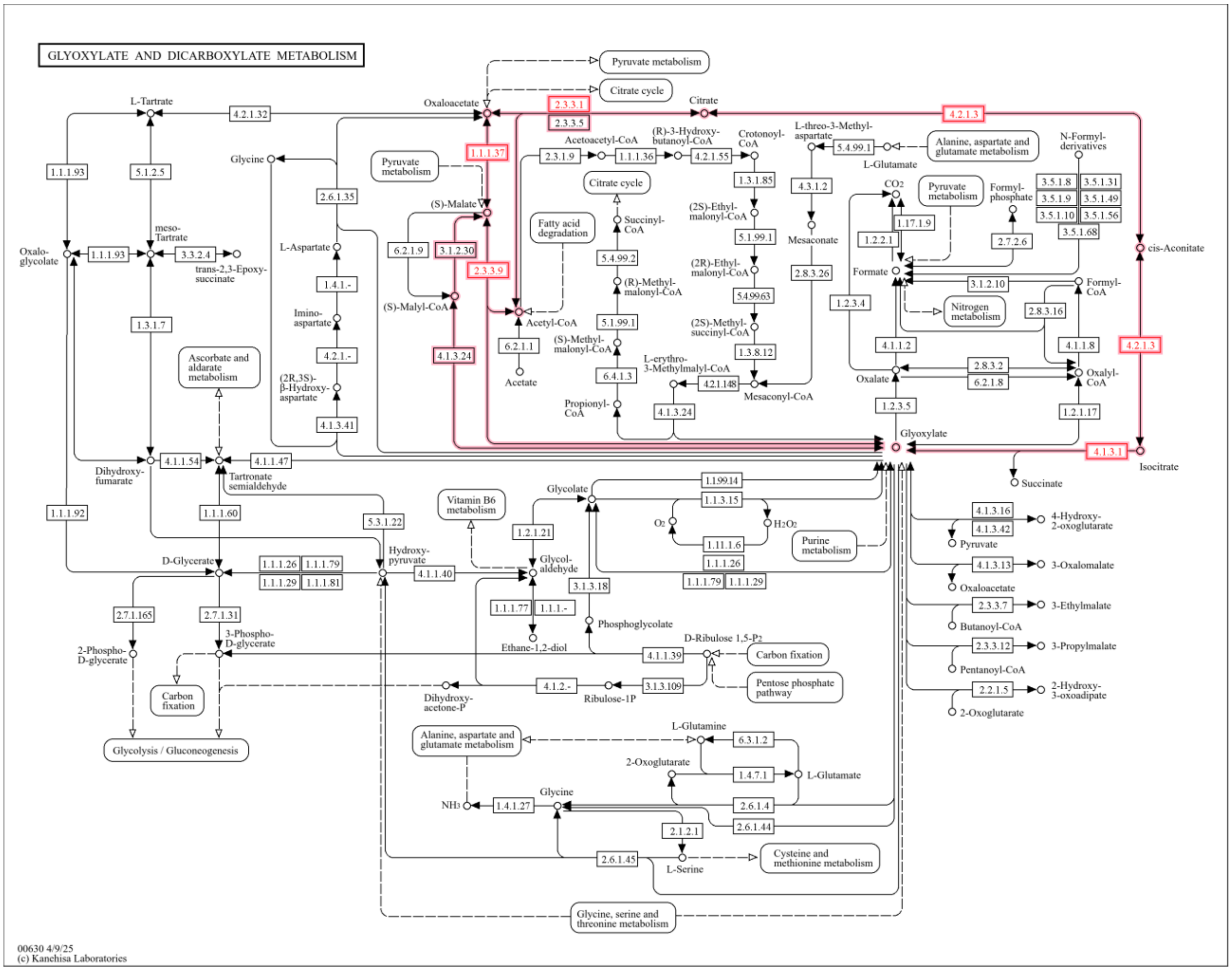

Supplementary Figure S1. Glyoxylate pathways diagram adapted from KEGG (map00630). Enzymes confirmed in *A. alstroemeriae* S6 are highlighted in red. The date (4/9/2025) indicates the last KEGG database update for this pathway.
